# Respiratory microbiota and radiomics features in the stable COPD patients

**DOI:** 10.1186/s12931-023-02434-1

**Published:** 2023-05-12

**Authors:** Rong Wang, Chunrong Huang, Wenjie Yang, Cui Wang, Ping Wang, Leixin Guo, Jin Cao, Lin Huang, Hejie Song, Chenhong Zhang, Yunhui Zhang, Guochao Shi

**Affiliations:** 1 Department of Pulmonary and Critical Care Medicine, the Affiliated Hospital of Kunming University of Science and Technology, the First People’s Hospital of Yunnan Province, Kunming, 650032 People’s Republic of China; 2 Medical School, Kunming University of Science and Technology, Kunming, 650500 People’s Republic of China; 3 Department of Pulmonary and Critical Care Medicine, Ruijin Hospital, Shanghai Jiao Tong University School of Medicine. Institute of Respiratory Diseases, Shanghai Jiao Tong University School of Medicine. Shanghai Key Laboratory of Emergency Prevention, Diagnosis and Treatment of Respiratory Infectious Diseases, Shanghai, 200025 People’s Republic of China; 4 Department of Radiology, Ruijin Hospital, Shanghai Jiao Tong University School of Medicine, Shanghai, 200025 People’s Republic of China; 5 Department of Pulmonary and Critical Care Medicine, the Third People’s Hospital of Kunshan, Suzhou, 215300 People’s Republic of China; 6 State Key Laboratory of Microbial Metabolism, School of Life Sciences and Biotechnology, Shanghai Jiao Tong University, Shanghai, 200240 People’s Republic of China

**Keywords:** COPD, Respiratory microbiota, Chest CT, Radiomics

## Abstract

**Backgrounds:**

The respiratory microbiota and radiomics correlate with the disease severity and prognosis of chronic obstructive pulmonary disease (COPD). We aim to characterize the respiratory microbiota and radiomics features of COPD patients and explore the relationship between them.

**Methods:**

Sputa from stable COPD patients were collected for bacterial 16 S rRNA gene sequencing and fungal Internal Transcribed Spacer (ITS) sequencing. Chest computed tomography (CT) and 3D-CT analysis were conducted for radiomics information, including the percentages of low attenuation area below − 950 Hounsfield Units (LAA%), wall thickness (WT), and intraluminal area (Ai). WT and Ai were adjusted by body surface area (BSA) to WT/$\sqrt{\text{B}\text{S}\text{A}}$ and Ai/BSA, respectively. Some key pulmonary function indicators were collected, which included forced expiratory volume in one second (FEV1), forced vital capacity (FVC), diffusion lung carbon monoxide (DLco). Differences and correlations of microbiomics with radiomics and clinical indicators between different patient subgroups were assessed.

**Results:**

Two bacterial clusters dominated by *Streptococcus* and *Rothia* were identified. Chao and Shannon indices were higher in the *Streptococcus* cluster than that in the *Rothia* cluster. Principal Co-ordinates Analysis (PCoA) indicated significant differences between their community structures. Higher relative abundance of *Actinobacteria* was detected in the *Rothia* cluster. Some genera were more common in the *Streptococcus* cluster, mainly including *Leptotrichia*, *Oribacterium, Peptostreptococcus*. *Peptostreptococcus* was positively correlated with DLco per unit of alveolar volume as a percentage of predicted value (DLco/VA%pred). The patients with past-year exacerbations were more in the *Streptococcus* cluster. Fungal analysis revealed two clusters dominated by *Aspergillus* and *Candida*. Chao and Shannon indices of the *Aspergillus* cluster were higher than that in the *Candida* cluster. PCoA showed distinct community compositions between the two clusters. Greater abundance of *Cladosporium* and *Penicillium* was found in the *Aspergillus* cluster. The patients of the *Candida* cluster had upper FEV1 and FEV1/FVC levels. In radiomics, the patients of the *Rothia* cluster had higher LAA% and WT/$\sqrt{\text{B}\text{S}\text{A}}$ than those of the *Streptococcus* cluster. *Haemophilus, Neisseria and Cutaneotrichosporon* positively correlated with Ai/BSA, but *Cladosporium* negatively correlated with Ai/BSA.

**Conclusions:**

Among respiratory microbiota in stable COPD patients, *Streptococcus* dominance was associated with an increased risk of exacerbation, *and Rothia* dominance was relevant to worse emphysema and airway lesions. *Peptostreptococcus*, *Haemophilus, Neisseria and Cutaneotrichosporon* probably affected COPD progression and potentially could be disease prediction biomarkers.

**Supplementary Information:**

The online version contains supplementary material available at 10.1186/s12931-023-02434-1.

## Background

Chronic obstructive pulmonary disease (COPD) is a complex heterogeneous disease characterized by chronic lung inflammation, persistent airflow limitation, and various clinical symptoms, such as shortness of breath, frequent coughing or wheezing, and excessive mucus production. [1–3] The latest data from WHO reported that COPD is the third leading cause of death worldwide, accounting for approximately 6% of all deaths worldwide. [4] It is imminent for us to find potential biomarkers as the therapeutic targets to better manage COPD and achieve precise medicine.

Previous studies have shown the existence of various microbiome in the human upper and lower respiratory tracts, including bacteria, fungi, and viruses, as well as their genomes and metabolites. [5] Microbial inhalation and respiratory clearance can maintain microbial homeostasis in the lung [6, 7]. Chronic respiratory diseases such as COPD, asthma, bronchiectasis, and Idiopathic pulmonary fibrosis (IPF) cause airway clearance dysfunction and lead to ecological dysbiosis of the respiratory microbiota, which in turn exacerbates the airway inflammation and affects disease progression. [8, 9]. It has been shown that the dysbiosis of respiratory microbiota in COPD patients was correlated with the inflammatory phenotype, risk of exacerbation, disease severity, and mortality. For instance, Dicker et al. found that the relative abundance of Proteobacteria and *Haemophilus* was higher in the airways of COPD patients with low peripheral blood eosinophils (< 100 cells/µl), and the relative abundance of Firmicutes and *Haemophilus* was positively associated with the 4 year-mortality. [10] Besides, COPD patients with high risk of exacerbation had reduced microbial alpha diversity, higher frequency of Proteobacteria and less *Streptococcus* in the airway microbiome, moreover, the relative abundance of Proteobacteria and *Neisseria* was negatively correlated with the ratio of forced expiratory volume in one second (FEV1) to forced vital capacity (FVC). [11] There is no doubt that the respiratory microbiota is closely associated with COPD.

Radiomics aims to convert digital medical images (e.g. chest CT) into mineable high-dimensional data and reveal the underlying pathophysiological information reflected in biomedical images through quantitative image analysis [12]. Chest CT is an important approach to assessing the disease in COPD patients. Advances in radiomics have allowed the high-throughput acquisition of numerous valuable quantitative imaging features from chest CT of COPD patients. [13, 14] For instance, the percentage of low attenuation area below − 950 Hounsfield Units (LAA%-950) can be calculated to assess the degree of emphysema, and wall thickness (WT), intraluminal area (Ai), and airway volume percent (AWV%) are used to evaluate the airway lesions. [15, 16] Assessing emphysema changes in COPD patients by chest CT could detect disease progression before pulmonary function declines. [17] Chest CT could find emphysema progression within 3 months in severe COPD patients. [18] It also has been found that emphysema and airway lesions of different severity are associated with survival prognosis, pulmonary function, and changes in COPD patients. Tanabe et al. reported that increased LAA% in COPD patients had the strongest correlation with an additional decrease in FEV1. [19] COPD patients with mixed imaging phenotypes (significant emphysema and airway lesions) and airway remodeling lesions with airway loss predominantly had worse survival prognoses. [20, 21] Both respiratory microbiomics and radiomics have been found to be relevant to COPD progression and disease severity, but the correlation between them is not yet clear. Understanding their correlation will not only help to understand the relationship between respiratory microbes and structural changes in the lung, but more importantly, may help to identify new therapeutic targets and develop more efficient clinical diagnostic and predictive models that integrate respiratory microbiomics and radiomics.

In this study, we aimed to characterize sputum bacterial and fungal microbiota in COPD patients. We also obtained the radiomics information through 3D-analysis of high-resolution chest CT and explored the relationships between respiratory microbiota and radiomics features in COPD patients. These integrated analyses are expected to help us gain a more comprehensive insight into the involvement of the respiratory microbiota and radiomics in COPD.

## Methods

### Study population

Clinical stable COPD patients were recruited from Ruijin Hospital, Shanghai Jiao Tong University School of Medicine, Shanghai, China. Written informed consent was obtained from all patients at the time of recruitment, and the study was approved by the Ethics Committee of Ruijin Hospital. All experiments were conducted in compliance and in line with the principles of the Declaration of Helsinki. Patients were enrolled according to the following criteria: (1) > 40 years old, (2) meeting the diagnostic criteria of COPD according to the guideline of the Global Initiative for Chronic Obstructive Lung Disease (GOLD) (FEV1/FVC% <70%, inhaled bronchodilators), (3) absence of exacerbation and systemic antibiotics using within the last 4 weeks before enrollment. Exacerbation was defined as an acute worsening of respiratory symptoms requiring additional treatment. [22, 23] The patients who were diagnosed with other respiratory diseases (e.g., asthma, tuberculosis, pneumonia, bronchiectasis, interstitial lung disease, lung cancer, etc.), who was treated with systemic steroids, immunosuppressive drugs, or systemic antibiotics within the last 4 weeks or for long-term were excluded. A total of 81 stable COPD patients were included in the study from July 2021 to February 2022.

### Spirometry and clinical data

All the patients underwent spirometry tests, [24] including FEV1, FVC, the ratio of FEV1 to FVC (FEV1/FVC), diffusion lung carbon monoxide single breath (DLco SB), DLco SB as a percentage of the predicted value (DLco SB%pred), DLco per unit of alveolar volume (DLco/VA), and DLco/VA as a percentage of the predicted value (DLco/VA%pred), etc. Other clinical data, including demographic data, smoking status, history of medication, exacerbations in the past 1 year, the Modified British Medical Research Council (mMRC) Questionnaire, and the COPD Assessment Test (CAT) Questionnaires, were collected.

### Sputum sample collection and processing

Spontaneous sputum samples were collected after rinsing the mouth at least thrice with 0.9% saline. Mucous fraction in sputum specimens for DNA extraction and sequencing were transported into sterile tubes and frozen immediately with dry ice and then stored at − 80 °C before analysis.

### Sputum DNA extraction and microbiota sequencing

DNA was extracted according to the instructions of the QIAamp DNA Microbiome Kit (Qiagen, Hilden, Germany) and stored at -20 °C for sequencing. PCR amplification was performed for quality control (QC) after DNA extraction. The PCR products were visualized after amplification using a 1.5% agarose gel to ensure successful amplification and correct band size. The 16 S rRNA and ITS genes were amplified, and libraries were constructed by TruSeq® DNA PCR-Free Sample Preparation Kit (Illumina, USA) targeting the V3-V4 hypervariable region of bacterial 16 S rRNA and the ITS1-ITS2 region of fungal, respectively. Double-end sequencing was performed using Illumina MiSeq (Illumina, USA) platform. The forward primer for amplifying the V3-V4 region was “338F: ACTCCTACGGGAGGCAGCAG” and the reverse primer was “806R: GGACTACHVGGGTWTCTAAT”. The forward primer for the amplification of the ITS1-ITS2 region was “ITS1F: CTTGGTCATTTAGAGGAAGTAA” and the reverse primer was “ITS2R: GCTGCGTTCTTCATCGATGC “.

### Bioinformatics analysis

Microbial sequencing data analysis was performed using Qiime2 (version 2020.2), R (version 3.3.1), Python (version 2.7), and Mothur (version 1.30). After sample splitting of the paired-end (PE) reads and quality control, the reads were filtered using Fastp (version 0.19.6), spliced according to the overlapping relationship between the PE reads using FLASH (version 1.2.7), and denoised to generate amplicon sequence variants (ASVs) using DADA2 algorithm. [25] The representative sequences of Amplicon Sequence Variant (ASV) of bacteria and fungi were taxonomy annotated by silva138/16s_bacteria and unite8.0/its_fungi databases, respectively, using the Naive Bayes classifier with a classification confidence level of 0.7. Jensen–Shannon divergence (JSD) distance algorithm was applied for sample colony typing analysis. [26, 27] Alpha diversity analysis was performed using mothur (v1.30.0) to obtain the Chao and Shannon indices, which were used to assess the microbial richness and diversity in the sputum samples. Principal Co-ordinates Analysis (PCoA) was performed based on the Weighted UniFrac distance to determine beta diversity. Microbial modularity analyses were conducted through the modularity function in Gephi software (Gephi 0.10).

### Three-dimensional (3D) CT analysis of the lungs

**Fig. 1:**
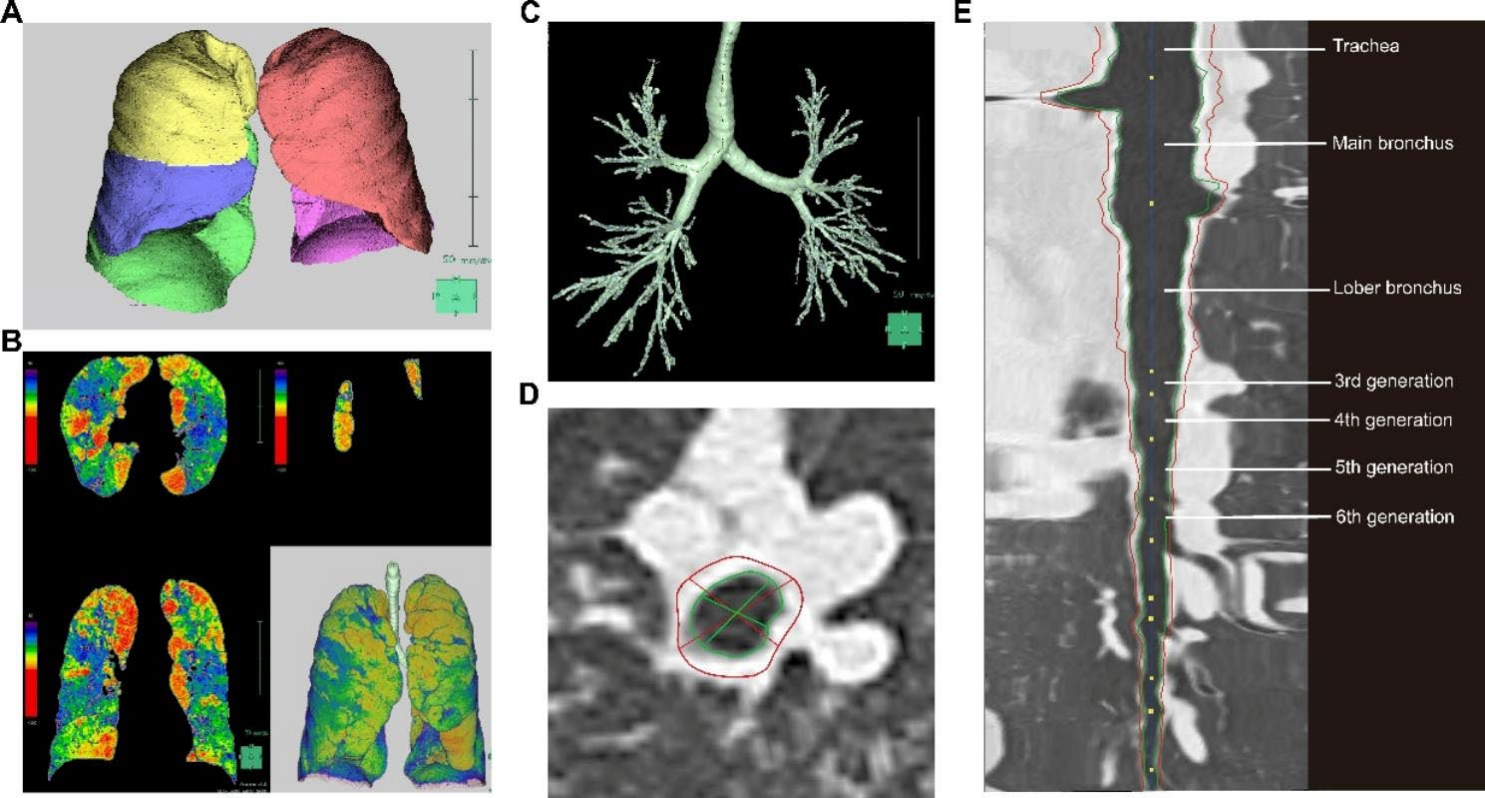
3D analysis of chest CT using SYNAPSE VINCENT. (**A**) Lung lobe extraction and segmentation. (**B**) Low attenuation area (LAA%) distribution analysis, the closer to red means more severe emphysema, the closer to blue means less severe emphysema. (**C**) 3D CT reconstruction image of the airway tree. (**D**) Cross-sectional images of selected airways, with the red line indicating the outer wall and outer diameter of the airway and the green line indicating the inner wall and inner diameter of the airway. (**E**) Longitudinal section image of the selected airway, yellow dots are automatically marked branch points

High-resolution Chest CT (at least 1.25 mm) scans from 28 patients were used to assess lung parenchyma by 3D analysis based on SYNAPSE VINCENT (Fuji Film, Tokyo, Japan) (Fig. 1) [28–30]. First, lung lobes were automatically extracted and segmented, and the percentage of low attenuation area below − 950 Hounsfield Units (LAA%) was calculated for the whole lung and each lung lobe. Afterward, bronchial pathways were obtained by 3D reconstruction and automatically mapped into multiplanar reconstructed images, and wall thickness (WT, mm) and intraluminal area (Ai, mm^2^) perpendicular to the long axis of the airway were automatically calculated. WT and Ai were measured at the midpoints of four levels (third generation [segmental], fourth generation [subsegmental], fifth generation, and sixth generation) of six bronchi in six segments of the right lung bronchi (B1, B2, B3, B8, B9, and B10). WT and Ai for each generation of bronchi were expressed as the mean of the six airways, and WT/$\sqrt{\text{B}\text{S}\text{A}}$ and Ai/BSA were obtained by adjusting with body surface area, respectively, to eliminate the potential effect of body size in each patient.

### Statistical analysis

Statistical analysis was performed using SPSS 25 (IBM Inc., Armonk, NY) and GraphPad Prism 9.0 (GraphPad Software, Inc., La Jolla, CA). Microbial sequencing data was analyzed using Qiime2 (version 2020.2) and R (version 3.3.1). Continuous data were presented as mean (standard deviation, SD) or median (interquartile range), and categorical data were expressed as numbers and percentages. The differences in clinical characteristics were compared by unpaired *t*-tests, Mann-Whitney u test, and chi-square test when needed. The comparisons of α-diversity indices and community compositions were analyzed using Wilcoxon rank-sum test with FDR for multiple testing adjustment. Between-group difference test for beta diversity was performed with ANOSIM. Spearman rank correlation coefficient with FDR-adjusted *P* value and Linear Regression were used to analyze the correlations between microbiota and their diversity, clinical indicators. Spearman correlations were calculated for the networks in modules with the FDR-adjusted *P* value. FDR *P* < 0.05 was considered statistically significant.

## Results

### Bacterial microbiota of sputum in stable COPD patients

Based on the QC results, 52 qualified DNA samples were available for 16 S rRNA gene sequencing among the 81 sputum specimens from 81 patients. Demographic and clinical characteristics information of the 52 patients were presented in Table S1 (in supplementary materials).

### Taxonomic characterization of bacterial microbiota

**Fig. 2:**
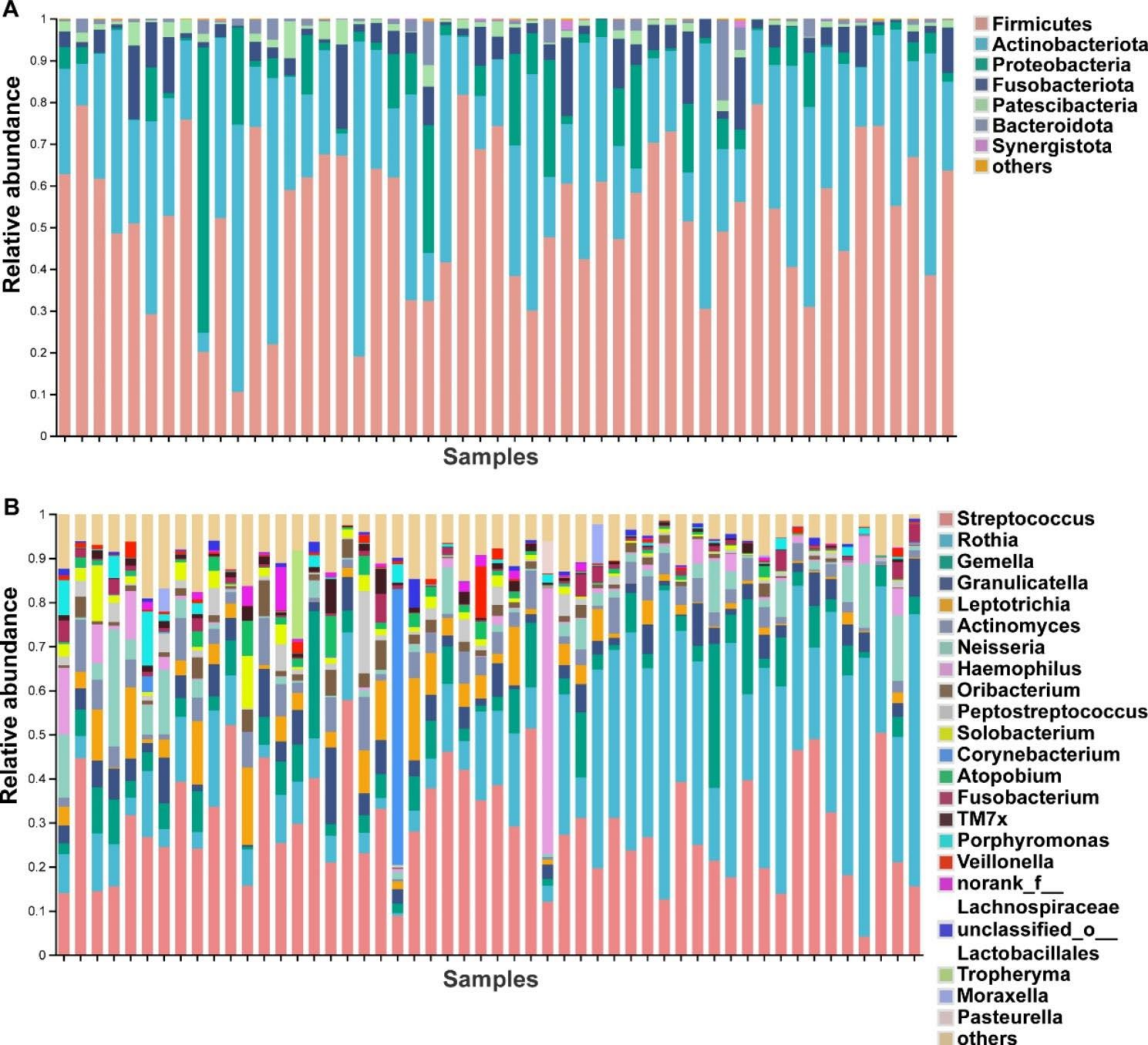
Composition of the respiratory bacterial community in 52 patients. (**A**) Community composition at the phylum level. (**B**) Community composition at the genus level

According to the lowest number of sequences, each bacterial sample was rarefied to 4463 sequences, representing 1393 bacterial ASVs. The top five bacteria at the phylum level were Firmicutes (53.21%), Actinobacteriota (29.99%), Proteobacteria (7.56%), Fusobacteriota (5.52%), Patescibacteria (1.75%) (Fig. 2A). The top five bacteria at the genus level were *Streptococcus* (29.32%), *Rothia* (22.31%), *Gemella* (6.74%), *Granulicatella* (4.68%), *Leptotrichia* (4.30%) (Fig. 2B).

**Fig. 3:**
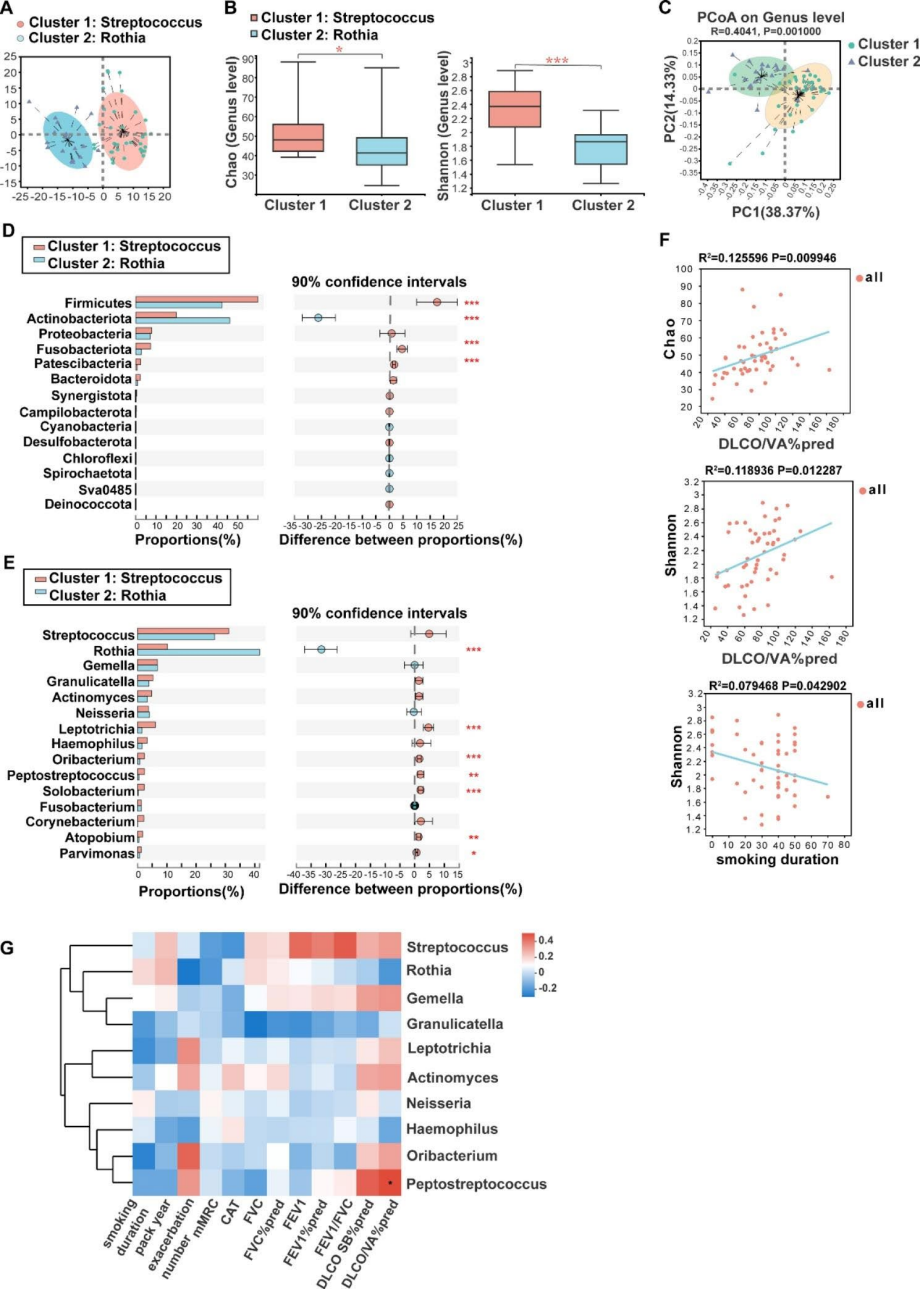
Comparison of alpha diversity and community composition between the *Streptococcus* cluster and the *Rothia* cluster. (**A**) JSD cluster stratification for sputum bacteria in 52 COPD patients, 1 was defined as *Streptococcus* cluster (n = 33), cluster 2 was defined as *Rothia* cluster (n = 19). (**B**) Comparison of alpha diversity (Chao and Shannon indices) between the two bacterial clusters. (**C**) PCoA analysis based on the Weighted UniFrac distance. (**D**) Significantly differing bacteria between the *Streptococcus* cluster and the *Rothia* cluster at the phylum level. (**E**) Significantly differing bacteria between the *Streptococcus* cluster and the *Rothia* cluster at the genus level. (**F**) DLco/VA%pred and smoking duration were associated with Chao and Shannon indices of bacteria by linear regression. (**G**) Some bacteria at the genus level were associated with clinical characteristics and pulmonary function based on Spearman correlation. Red indicates positive correlation; blue indicates negative correlation(* 0.01 ≤ FDR *P*<0.05, ** 0.001 ≤ FDR *P*<0.01, *** FDR *P* < 0.001).

We performed JSD cluster stratification for sputum bacteria and identified two clusters in our patients. Cluster 1 had the highest-level of genus *Streptococcus* (*Streptococcus* cluster, n = 33), and cluster 2 was made up of highly abundant *Rothia* (*Rothia* cluster, n = 19) (Fig. 3A). Venn analysis showed the two clusters shared 106 bacteria and 79 fungi (Fig S1 C, D, in supplementary materials). The comparisons of clinical characteristics of the two clusters revealed that the DLco/VA%pred was higher in the *Streptococcus* cluster than that in patients of the *Rothia* cluster (82.911 ± 25.069

vs. 64.471 ± 26.144), while no differences were found in DLco SB%pred, FEV1%pred, FEV1/FVC% and GOLD classifications between the two clusters (Table S2, in supplementary materials). Furthermore, the proportion of patients with exacerbations in the past 1 year was higher in the Streptococcus cluster than in the Rothia cluster (54.5% vs 26.3%) (Table S2, in supplementary materials).

Comparisons of alpha diversity unveiled higher bacterial richness and diversity in the *Streptococcus* cluster than that in the *Rothia* cluster, as evidenced by the higher Chao and Shannon indices (Fig. 3B). PCoA showed significant differences in the bacterial composition between two clusters (ANOSIM, R = 0.377, *P* = 0.001) (Fig. 3C). Then, we compared the compositional differences between the two clusters. At the phylum level, the relative abundance of Firmicutes, Fusobacteria, and Patescibacteria in the *Streptococcus* cluster was significantly higher than that in the *Rothia* cluster, while the relative abundance of Actinobacteriota was higher in the *Rothia* cluster (Fig. 3D). At the genus level, six genera were more common in the *Streptococcus* cluster than that in the *Rothia* cluster, including *Leptotrichia*, *Oribacterium, Peptostreptococcus, Solobacterium, Atopobium*, and *Parvimonas* (Fig. 3E). Fungal composition at the phylum level and genus level in these two bacterial clusters was shown in Fig.S1 A and B (in supplementary materials). We also compared the fungal composition between the *Streptococcus* and *Rothia* clusters. The fungus that differed significantly between the two clusters at the phylum level was Rozellomycota (Fig.S1 E, in supplementary materials). There was no significant difference in fungal composition between the two clusters at the genera level (Fig.S1 F, in supplementary materials).

### Correlations between bacterial microbiota and clinical indices

Furthermore, we sought to investigate the relationships between bacterial microbiota and clinical traits in the two clusters. Through linear regression analysis, we observed the relationships between clinical indicators and bacterial alpha diversity. The data showed that Chao and Shannon indices of bacteria were positively correlated with DLco/VA%pred, indicating that higher bacterial richness and diversity were associated with better diffusion function (Fig. 3F). Shannon index was negatively correlated with smoking duration, indicating that smoking may decrease bacterial diversity in the airway (Fig. 3F). In addition, we selected genus species with top10 relative abundance and some clinical and pulmonary function indicators to perform Spearman correlation analysis with FDR-adjusted *P* values. The results showed that *Peptostreptococcus* was positively correlated with DLco/VA%pred (R = 0.401, FDR *P* < 0.05) (Fig. 3G).

### Fungal microbiota of sputum in stable COPD patients

#### Taxonomic characterization of fungal microbiota

Based on the QC results, 30 qualified DNA samples were available for ITS sequencing among the 81 sputum specimens from 81 patients. Demographic and clinical characteristics information of the 30 patients were presented in Table S3 (in supplementary materials).

#### Taxonomic characterization of fungal microbiota

**Fig. 4:**
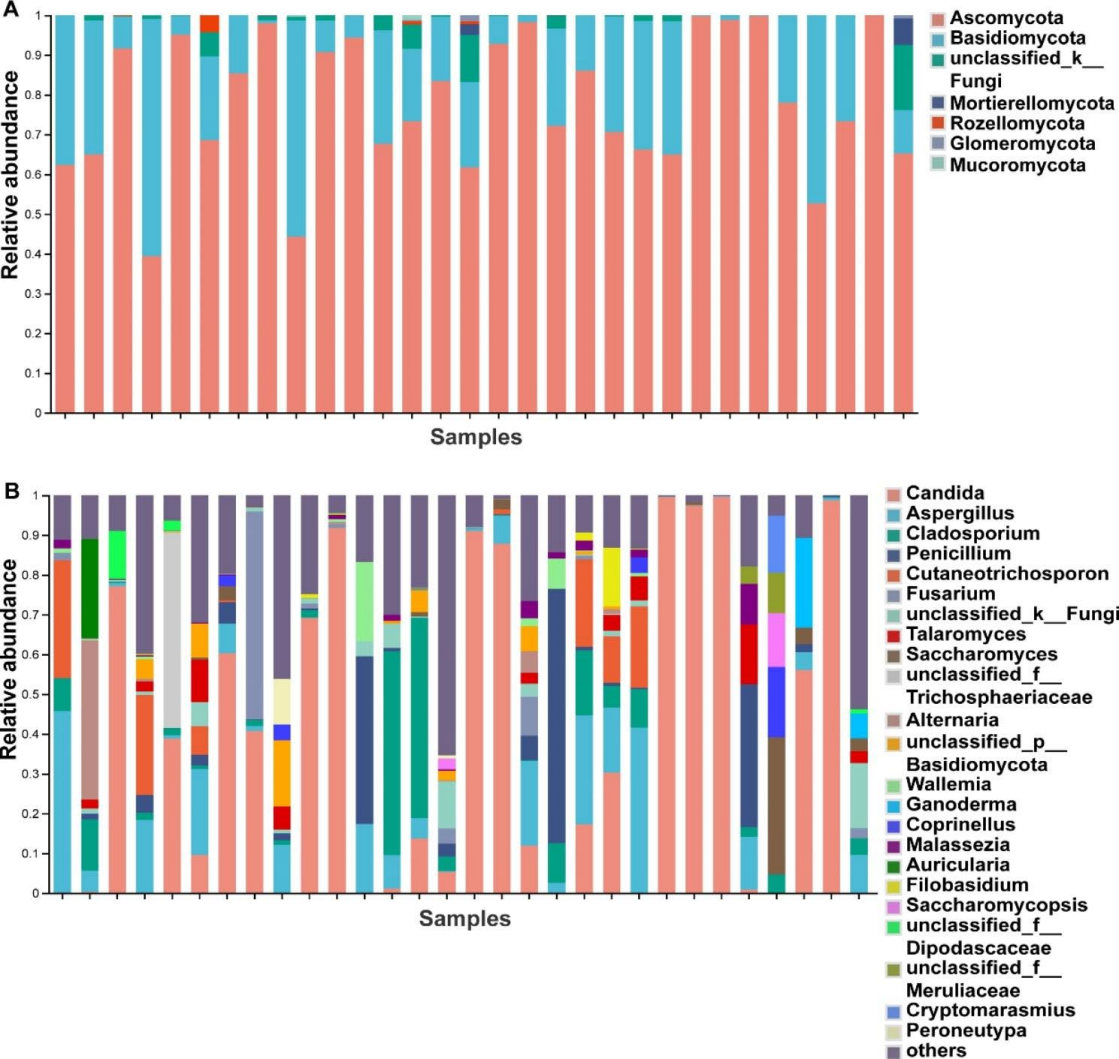
Composition of the respiratory fungi community in 30 patients. (**A**) Community composition at the phylum level. (**B**) Community composition at the genus level

According to the lowest number of sequences, each fungal sample was rarefied to 31,812 sequences, representing 970 fungal ASVs. The top two fungi at the phylum level included Ascomycota (78.0%), Basidiomycota (19.4%) (Fig. 4A). The top five fungi at the genus level were *Candida* (36.6%), *Aspergillus* (9.7%), *Cladosporium* (6.3%), *Penicillium* (5.8%), and *Cutaneotrichosporon* (3.9%) (Fig. 4B).

**Fig. 5:**
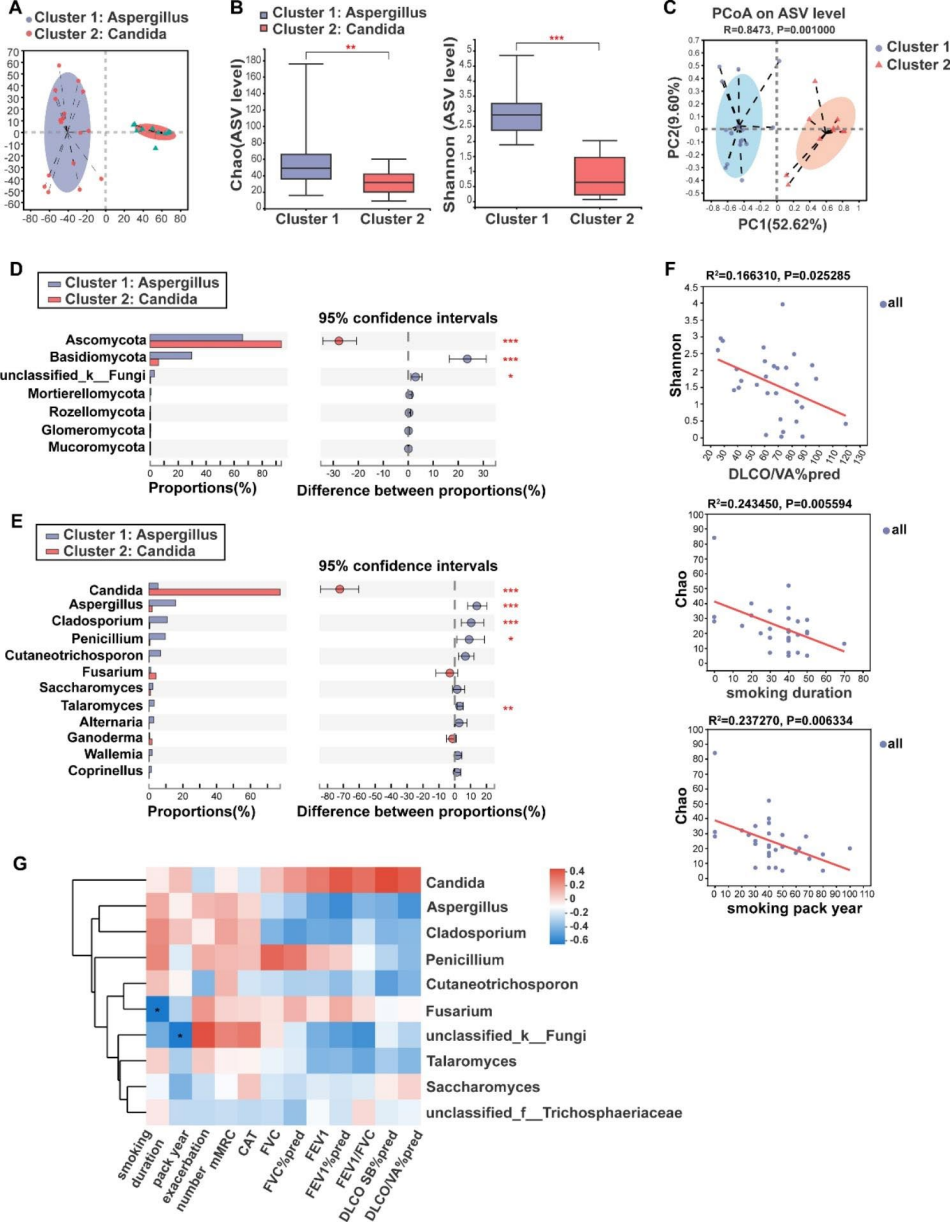
Comparison of alpha diversity and community composition between *Aspergillus* cluster and *Candida* cluster. (**A**) JSD cluster stratification for sputum fungi in 30 COPD patients, cluster 1 was defined as *Aspergillus* cluster(n = 17), cluster 2 was defined as *Candida* cluster(n = 13). (**B**) Comparison of alpha diversity (Chao and Shannon indices) between the two fungal clusters. (**C**) PCoA analysis based on the Weighted UniFrac distance. (**D**) Significantly differing fungi between *Aspergillus* cluster and *Candida* cluster at the phylum level. (**E**) Significantly differing fungi between *Aspergillus* cluster and *Candida* cluster at the genus level. (**F**) DLco/VA%pred, smoking duration, and smoking pack year were associated with Chao and Shannon indices of bacteria by linear regression. (**G**) Some fungi at the genus level were associated with clinical characteristics and pulmonary function based on Spearman correlation. Red indicates positive correlation; blue indicates negative correlation(* 0.01 ≤ FDR *P*<0.05, ** 0.001 ≤ FDR *P*<0.01, *** FDR *P* < 0.001)

Similarly, JSD cluster stratification for the fungi revealed two clusters , cluster 1 was dominated by *Aspergillus* (*Aspergillus* cluster, n = 17), and cluster 2 was dominated by *Candida* (*Candida* cluster, n = 13) (Fig. 5A). Venn analysis showed the two clusters shared 84 fungi and 87 bacteria (Fig S2 C, D, in supplementary materials). There were significant variations in their major pulmonary function indicators (Table S4). The *Candida* cluster had significantly higher FEV1(1.52 ± 0.36 vs. 1.20 ± 0.41) and FEV1/FVC (55.39 ± 8.77 vs. 46.87 ± 12.05) levels than the *Aspergillus* cluster (Table S4). The *Candida* cluster also exhibited higher FEV1%pred, FVC%pred, DLco SB%pred, and DLco/VA%pred levels than the *Aspergillus* cluster, although they did not reach statistical difference (Table S4). There were more patients with mild to moderate airflow limitation i*n the Candida* cluster and more patients with severe airflow limitation in the *Aspergillus* cluster (Table S4, in supplementary materials).

Alpha-diversity comparisons showed that the Chao and Shannon indices of the *Aspergillus* cluster were significantly higher than that in the *Candida* cluster (Fig. 5B). PCoA showed significant differences in fungal community composition between the two clusters (ANOSIM, R = 0.843, p = 0.001) (Fig. 5C). Then we compared the relative abundances of fungal phyla between the two clusters. The relative abundance of Basidiomycota was higher in the *Aspergillus* cluster and the proportion of Ascomycota in the *Candida* cluster was higher (Fig. 5D). At the genus level, the relative abundances of *Cladosporium*, *Penicillium*, etc. were higher in the *Aspergillus* cluster (Fig. 5E). The major bacteria in these two fungal clusters at the phylum level and genus level were presented in the Fig. S2 A and B (in supplementary materials). We compared the bacterial composition between the *Aspergillus* and *Candida* clusters. The bacterial species differing at the phylum and genera levels were Proteobacteria, *Streptococcus* and *Neisseria* (Fig. S2 E, F, in supplementary materials). In addition, we analyzed bacteria-fungi correlations between the two clusters, and the results showed bacteria-fungi associations, such as close connections between *Haemophilus*, *Neisseria*, *Moraxella*, *Plectosphaerella*, *Saccharomycopsis*, *Wickerhamomyces*, etc. (pink module) in *Aspergillus* cluster (Fig. S3, in supplementary materials), as also as between *Psathyrella*, *Saccharomyces*, *Phaeosphaeria*, *Peniophora*, *Leptotricchia*, etc. (blue module) in *Candida* cluster (Fig S4, in supplementary materials).

### Correlations between fungal microbiota and clinical indices

Furthermore, we explored the association between fungal microbiota and clinical indices of these COPD patients. The results revealed that the Shannon index was negatively correlated with DLco VA%pred, which meant that higher fungal diversity was associated with worse diffusion function (Fig. 5F). Chao index was negatively correlated with smoking duration and smoking pack years, indicating that smoking could reduce the fungal richness (Fig. 5F). Additionally, Spearman correlation analysis showed that *Fusarium* was negatively associated with smoking duration (R= -0.537, FDR *P* < 0.05) (Fig. 5G).

### Radiomics features in stable COPD patients and the correlations between radiomics and respiratory microbiota

As mentioned previously, radiomics involving 3D CT analysis of chest CT could calculate LAA% for emphysema, WT and Ai for airway lesions, which were adjusted for BSA as WT/√BSA and Ai/BSA, respectively.

**Fig. 6:**
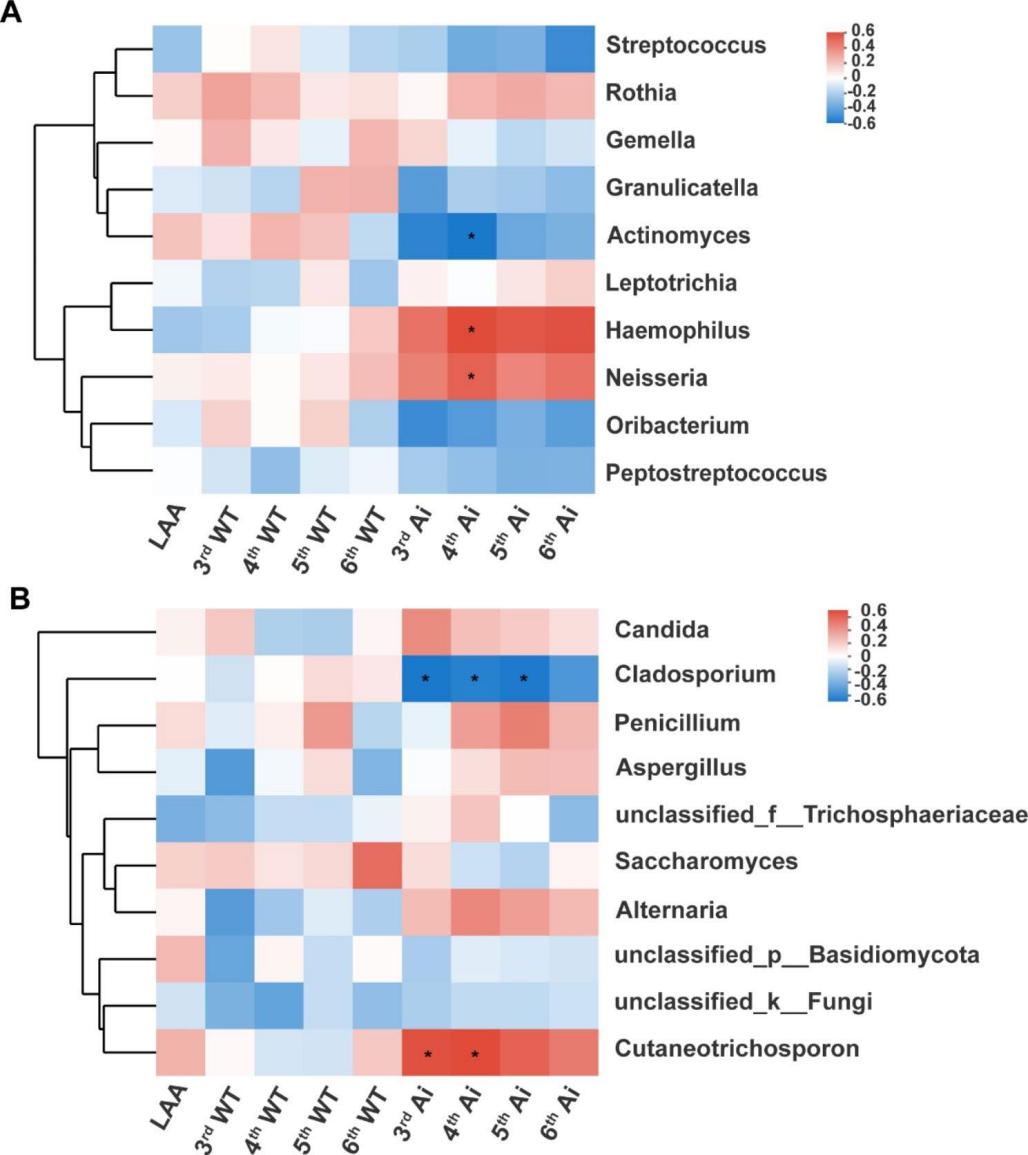
Spearman correlation analysis for respiratory microbiota and radiomics features. (**A**) The correlations between radiomics features and respiratory bacterial microbiota. (**B**) The correlations between radiomics features and respiratory fungal microbiota. (* FDR *P*<0.05)

In bacterial microbiota, 28 patients performed chest CT (at least 1.25 mm), including 17 patients from the *Streptococcus* cluster and 11 patients from the *Rothia* cluster. It revealed that the *Rothia* cluster had higher LAA% in the whole lung and left upper lobe than the *Streptococcus* cluster (39.49 ± 9.98 vs. 29.36 ± 13.54, 42.03 ± 10.96 vs. 29.66 ± 15.08, respectively) (Table S5). The *Rothia* cluster displayed higher WT/$\sqrt{\text{B}\text{S}\text{A}}$ than *Streptococcus* cluster in 3^rd^ (1.16 ± 0.27 vs. 0.93 ± 0.28), 4^th^ (1.16 ± 0.27 vs. 0.93 ± 0.28), 6^th^ generation bronchi (0.67 ± 0.12 vs. 0.59 ± 0.09) (Table S5). However, there were no significant differences in Ai/BSA of the 3^rd^ to 6^th^ generation bronchi between the two clusters (Table S5, in supplementary materials). The results indicated that emphysema and airway lesions were more severe in the *Rothia* cluster than that in the *Streptococcus* cluster. Furthermore, we sought to investigate the correlations between radiomics and respiratory bacterial microbiota. Spearman correlation analysis showed that the relative abundances of *Haemophilus* (R = 0.532, FDR *P* < 0.05) and *Neisseria* (R = 0.465, FDR *P* < 0.05) were positively correlated with Ai/BSA of the 4^th^ generation bronchus (Fig. 6A). In contrast, the relative abundance of *Actinomyces* was inversely correlated with Ai/BSA of the 4^th^ generation bronchus (R=-0.510, FDR *P* < 0.05) (Fig. 6A).

In fungal microbiota, 3D-CT analysis was performed for 7 patients of the *Aspergillus* cluster and 8 patients of the *Candida* cluster. There were no significant differences in LAA% in the whole lung and each lung lobe, and WT/$\sqrt{\text{B}\text{S}\text{A}}$ in each generation bronchus between the *Aspergillus* cluster and *Candida* cluster (Table S6). The *Candida* cluster had a larger Ai/BSA of 3^rd^ generation bronchus than the *Aspergillus* cluster (38.26 ± 11.25 vs. 25.52 ± 9.53), implying that patients of the *Aspergillus* cluster might have more severe airway lesions than patients of the *Candida* cluster (Table S6, in supplementary materials). Next, we also performed a Spearman correlation analysis of fungi microbiota and radiomics features. The results showed that *Cutaneotrichosporon* was positively correlated with Ai/BSA of the 3^rd^ (R = 0.630, FDR *P* < 0.05) and 4^th^ (R = 0.651, FDR *P* < 0.05) generation bronchi (Fig. 6B). Conversely, *Cladosporium* was negatively correlated with Ai/BSA of the 3^rd^ to 5^th^ generation bronchi (R=-0.699, R=-0.661, R=-0.693, FDR *P* < 0.05) (Fig. 6B).

## Discussion

In general, we identified two clusters of patients according to the dominant genus in both bacterial microbiota and fungal microbiota using the JSD method, and these clusters differed in bacterial and fungal diversity, community composition, and clinical characteristics. For instance, the *Streptococcus* cluster showed higher bacterial richness and diversity than the *Rothia* cluster. The *Aspergillus* cluster showed higher bacterial richness and diversity than the *Candida* cluster. There was an increased number of patients with exacerbation in the *Streptococcus* cluster and patients with higher levels of pulmonary ventilation in the *Candida* cluster. Moreover, we firstly investigated the integration of radiomics and respiratory microbiomics in COPD patients, we also characterized the features of radiomics between two bacterial and fungal clusters, respectively. In addition, distinct associations between radiomics and bacterial microbiota, and fungal microbiota were found in COPD patients.

The respiratory microbiota of COPD patients was previously categorized into different microbial ecological subtypes based on the presence of certain dominant genera, which was essentially a microbial ecological dysbiosis. [10, 31] Overgrowth of a particular bacteria might be an important factor driving the ecological dysbiosis of respiratory microbes and potentially altering the pulmonary disease state. [32, 33] COPD patients are prone to airway microbial dysbiosis, usually with one or more microbes as the dominant species. Wang et al. found that there were persistent homeostatic microbiota group and other three groups dominated by *Haemophilus*, *Moraxella* and *Streptococcus*, respectively, in COPD patients with a neutrophilic inflammatory phenotype, both in the stable and exacerbation phases. [31] Other dysbiosis subtypes with Proteobacte and Firmicutes predominating, respectively ,were also identified in COPD patients. [10] We therefore hypothesize that such cluster formation is the result of respiratory microbial ecological dysregulation and is associated with disease state or inflammation, which might interact with each other. Through cluster analysis, we identified the *Streptococcus* cluster and the *Rothia* cluster among the respiratory bacteria of these COPD patients, which differed in terms of microbial composition and diversity as well as clinical characteristics. Our data showed that there were more patients with exacerbations that occurred in the past 1 year in the *Streptococcus* cluster than those in the *Rothia* cluster. Similar to our results, Dicker and colleagues observed that there were more frequent exacerbators and mMRC ≥ 2 patients with a higher mortality risk in the *Streptococcus* dominant subtype compared to the balanced profile among 252 COPD participants. [10] In contrast, Ren et al. found a lower exacerbation frequency in patients with *Streptococcus* and *Rothia*-enriched community subtypes, and they considered that pulmonary colonization by certain specific bacteria (e.g., *Streptococcus spp.*, *Rothia spp.*) might have a protective effect against progression. [34] There were some discrepancies between the above research and ours, which probably related to the type of samples collected, the sequencing method and the differences in the conditions of the patients included. Therefore, these data suggested that *Streptococcus* dominant community subtypes were correlated with COPD exacerbations, which was possibly used to predict the risk of exacerbation. However, the reasons behind this correlation remain unclear. Wang et al. showed that *Streptococcus* probably indirectly triggered CXCL8/IL-8 excess production through pulmonary microbiota dysbiosis, while elevated sputum CXCL8/IL-8 levels correlated with increased severity of COPD. [32, 35, 36]*Streptococcus pneumoniae* might mediate impaired TLR2 signaling of alveolar macrophages in patients with frequent exacerbations of COPD. [37] We hypothesized that *Streptococcus* could affect the immune response in COPD patients, promote an inflammatory response in the lungs of COPD patients and thus increase the risk of exacerbation. A larger sample size and long-term follow-up were needed to confirm it.

In addition, we found that not only dominant genera but also some low-abundance key genera in might be involved in the disease status of COPD. For example, low-abundance *Peptostreptococcus* was significantly positively correlated with diffusion function, whose relative abundance was higher in the *Streptococcus* cluster than that in the *Rothia* cluster. Thus, we speculated that the higher dispersion function of the *Streptococcus* cluster was partly related to *Peptostreptococcus*. Previous paper revealed that the relative abundance of *Peptostreptococcus* was reduced in the airways of COPD patients who failed in anti-infective treatment, which was also adversely correlated with the length of stay of AECOPD patients. [38] It might suggest that the reduction in *Peptostreptococcus* was involved in poor COPD disease prognosis. Moreover, earlier work has shown that metabolites produced by microbiota can have a crucial impact on both mucosal and circulating leukocytes. [39] In inflammatory bowel disease (IBD) studies, some species of *Peptostreptococcus*, such as *Peptostreptococcus russellii*, could promote intestinal epithelial barrier function and reduce the inflammatory response through the production of the tryptophan metabolite indoleacrylic acid (IA). [40] Another *Peptostreptococcus* specie, *Peptostreptococcus anaerobius*, has instead been found to be involved in the promotion of colorectal carcinogenesis. [41, 42] In contrast, in studies of non-small cell lung cancer (NSCLC), Peptostreptococcaceae, as an intestinal commensal, was more abundant in healthy control populations than in NSCLC patients. [43] Thus, it appears that different *Peptostreptococcus* species might perform beneficial or harmful roles in different diseases. It was interesting and necessary to further explore whether some beneficial *Peptostreptococcus* species could influence the inflammatory response in COPD by producing beneficial metabolites or other pathways in the lungs, and what the beneficial *Peptostreptococcus* are.

Owing to the low abundance of fungi in the respiratory tract in healthy individuals and COPD patients, and lower sensitivity of the detection method in our study, fewer patients with qualified sequence were eventually enrolled for fungal microbiota analysis than those for bacterial microbiota analysis. [44, 45] We identified the *Aspergillus* cluster and the *Candida* cluster with distinct microbial characteristics. Consistent with other research, we found that increased abundance of *Aspergillus* in the respiratory tract was associated with more severe disease, yet *Candida* with better disease status. Our data showed the *Aspergillus* cluster had worse pulmonary function levels and GOLD classification. Prior researches have found that not only did COPD patients with positive *Aspergillus* cultures had increased sputum inflammatory cells, but also the incidence of acute exacerbations in the previous 1 year was associated with an increase in positive *Aspergillus* isolates. [46]Patients with a dominance of *Aspergillus*, *Curvularia* and *Penicillium* had more frequent acute exacerbations and higher mortality. [47] Moreover, it was demonstrated that *Aspergillus*-sensitized COPD patients had significantly more exacerbations and hospital exacerbations, poorer lung function, and a worse prognosis. [48]*Aspergillus* was associated with worse disease, which might be related to the increased susceptibility of COPD patients to invasive aspergillosis. [49]*Aspergillus* caused a spectrum of clinical symptoms by causing invasive aspergillosis or allergic sensitization, depending on the immune status of the host. [50] The recent studies further showed that increased disease severity of COPD patients was related to fungal sensitization, which might be associated with neutrophilic influx and neutrophil extracellular trap (NET). [51] Moreover, we found that *Candida* was associatied with milder disease and better pulmonary ventilation function, which was inconsistent with pioneer research suggesting that *Candida* colonization of the lower respiratory tract was associated with 180-day exacerbation recurrence rate and 1-year mortality in patients with AECOPD [52]. Given the different disease states of patients, we hypothesized that *Candida* might have different effects on COPD patients in the stable and exacerbation phases. Taken together, our findings supported that *Aspergillus* and *Candida* could be used to distinguish different disease phenotypes, and they were associated with acute exacerbation risk and severity of disease. However, the causal relationship and specific mechanisms of them with COPD disease progression still need more research.

Radiomics has shown a critical role in the diagnosis and disease risk prediction for COPD. For example, decreased LAA% of chest CT quantification can lead to an increased risk of exercise hypoxia in COPD patients. [53] Airway remodeling lesions defined by chest CT were independently associated with decreased 6-minute walk distance, respiratory quality of life, and pulmonary function. Patients with predominantly lost airways had lower survival rates than patients with predominantly narrowed airways. [21] The combination of artificial intelligence (AI) technology and deep learning algorithms with chest imaging has greatly enhanced the efficacy of radiomics for disease risk prediction. Convolutional Neural Network (CNN) models not only predicted mortality in COPD patients based on a six-minute walk distance and high-throughput extraction of chest CT features but also enabled accurate diagnosis of COPD with only chest CT data. [54, 55] In our study, radiomics was performed to evaluate the emphysema and airway wall thickening in COPD, based on that, we integrated radiomics and microbiomics to explore the associations between airway microbiota and pulmonary structural damage in COPD. Our data revealed that emphysematous lesions and airway lesions were both more severe in the *Rothia* cluster than that in the *Streptococcus* cluster, which suggested that *Rothia*-enriched COPD patients probably had more severe pulmonary structural damage and airway remodeling. Similarly, the *Aspergillus* cluster had higher LAA% and smaller Ai than the *Candida* cluster, although the differences were not statistically significant. It reflected that *Aspergillus* dominance also might represent more severe emphysema and airway lesions. However, Engel et al. study showed that COPD patients with higher relative abundance of *Streptococcus* had significant CT-detectable emphysema and airway lesions, whereas these CT changes were not detected in patients with higher relative abundance of *Prevotella*. [56] The correlations between these dominant microbiota and emphysema or airway lesions are not clear, and more researches are therefore needed to clarify the question.

Bronchiectasis and COPD are two diseases with overlapping clinical features that can be co-diagnosed as “COPD-bronchiectasis association”, where the neutrophilic inflammation, differential mucin expression and gram-negative bacterial infection were the prominent features. [57] As a co-morbidity of COPD, the average prevalence of bronchiectasis in COPD patients is 54.3%, with a range of 25.6–69%. [58] Thus it can be assumed that bronchiectasis is a frequent pathological phenotype in the natural course of COPD. In our study, *Haemophilus*, *Neisseria* and *Cutaneotrichosporon* were found to be positively correlated with intraluminal area (Ai) in some airway generations, suggesting that these bacteria in the airway may be a cause of bronchiectasis in COPD patients. Of these bacteria, *Haemophilus influenzae* from genus *Haemophilus* has also been shown to promote the inflammatory response, which is one of the essential pathological mechanisms of COPD. [59, 60] Li et al. combined human cohorts, next-generation sequencing, systems biology and animal models to reveal a bronchodilator bacterial group defined by the presence of *Neisseria* spp., which identified *Neisseria* as a potential pathobiont of bronchiectasis. [61]*Cutaneotrichosporon*, an opportunistic fungus, has been found in respiratory specimens from patients with cystic fibrosis. [62] Consequently, *Haemophilus*, *Neisseria* and *Cutaneotrichosporon* showed tendencies to be relevant to bronchiectasis in COPD, whether by causing an inflammatory response or directly contributing to bronchodilator lesions. Therefore, we speculated that the positive correlations of these genera with Ai might be early signals for the airway dilatation in COPD patients. More prospective follow-up studies are needed later to test this conjecture.

There were some limitations to our research. Firstly, the sample size is small. Secondly, the sputum naturally coughed up by the patients might not be fully representative of lower respiratory tract microbiota in comparison to induced sputum or bronchoalveolar lavage fluid (BALF). Thirdly, the cross-sectional nature of our study was unable to depict the dynamic characteristics of respiratory microbiota during the disease process. In addition, the 16 S rRNA gene sequencing and ITS sequencing approach with a short read-length sequencing could not specify the species level, missing the real target microbes with significant differences observed in the present study. In contrast, metagenomic sequencing offsets these disadvantages, such as enhancing microbial species detection, increasing diversity testing and predicting gene function, and improving species detection accuracy. [63] Therefore, a large-scale study screening COPD patients in different disease stages with metagenomic sequencing is deserved to further delineate the role of respiratory microbiota in COPD initiation and development and characterize the associations between respiratory microbiota and radiomics in COPD. At last, our cross-sectional results were relatively limited in terms of clarifying the causal relationships between respiratory microbiota and disease characteristics, and long-term follow-up research is needed in the future.

## Conclusion

In summary, our research identified different microbiota clusters in airway bacteria and fungi of stable COPD patients. We also demonstrated that different microbial clusters and key genera in the respiratory microbiota were associated with distinct disease features, including exacerbation, pulmonary function, and lung structural lesions. Integration of respiratory microbiomics and radiomics for disease risk assessment and clinical prediction of COPD will be an attractive direction in the future.

## Electronic supplementary material

Below is the link to the electronic supplementary material.

Supplementary Material 1: **Table S1.** Characteristics of patients with respiratory bacteria sequencing (N = 52). **Table S2.** Comparison of clinical characteristics and pulmonary function between *Streptococcus* cluster and *Rothia* cluster. **Table S3.** Characteristics of patients with respiratory fungi sequencing (N = 30). **Table S4.** Comparison of clinical characteristics and pulmonary function between Aspergillus cluster and Candida cluster. **Table S5.** Comparison of the radiomics features in patients of two bacterial clusters. **Table S6.** Comparison of the radiomics features in patients of two fungal clusters. **Fig S1.** Fungi in the Streptococcus and Rothia clusters. **Fig S2.** Bacteria in the *Aspergillus* and *Candida* clusters. **Fig S3.** Bacteria– fungi, bacteria– bacteria, and fungi– fungi connections in *Aspergillus* cluster. **Fig S4.** bacteria– fungi, bacteria– bacteria, and fungi– fungi connections in Candida cluster.

## Data Availability

Sequence data are available at the National Center for Biotechnology Information Sequence Read Archive with accession number PRJNA938570.

## Abbreviations

AI: Artificial intelligence
Ai: Intraluminal area
ASV: Amplicon Sequence Variant
AWV: Airway volume
BALF: Bronchoalveolar lavage fluid
BSA: Body surface area
CAT: The COPD Assessment Test
CNN: Convolutional Neural Network
COPD: Chronic obstructive pulmonary disease
CT: Computed tomography
DLco SB: Diffusion lung carbon monoxide single breath
DLco/VA: Diffusion lung carbon monoxide per unit of alveolar volume
DLco: Diffusion lung carbon monoxide
FEV1: Forced expiratory volume in one second
FVC: Forced vital capacity
GOLD: The Global Initiative for Chronic Obstructive Lung Disease
ICS: Inhaled corticosteroids
IPF: Idiopathic pulmonary fibrosis
ITS: Fungal Internal Transcribed Spacer
JSD: Jensen–Shannon divergence
LAA%: The percentages of low attenuation area below − 950 Hounsfield Units
mMRC: The Modified British Medical Research Council
PCoA: Principal Co-ordinate Analysis
WT: Wall thickness
