# Supplementary material for: Respiratory microbiota and radiomics features in the stable COPD patients
Source: Respir Res. 2023 May 12;24:131. doi: 10.1186/s12931-023-02434-1 (PMC10176953; doi:10.1186/s12931-023-02434-1)
Supplement: Supplementary file 1 — Supplementary Material 1: Table S1. Characteristics of patients with respiratory bacteria sequencing (N = 52). Table S2. Comparison of clinical characteristics and pulmonary function between Streptococcus cluster and Rothia cluster. Table S3. Characteristics of patients with respiratory fungi sequencing (N = 30). Table S4. Comparison of clinical characteristics and pulmonary function between Aspergillus cluster and Candida cluster. Table S5. Comparison of the radiomics features in patients of two bacterial clusters. Table S6. Comparison of the radiomics features in patients of two fungal clusters. Fig S1. Fungi in the Streptococcus and Rothia clusters. Fig S2. Bacteria in the Aspergillus and Candida clusters. Fig S3. Bacteria– fungi, bacteria– bacteria, and fungi– fungi connections in Aspergillus cluster. Fig S4. bacteria– fungi, bacteria– bacteria, and fungi– fungi connections in Candida cluster. [file 12931_2023_2434_MOESM1_ESM.docx]

**Supplementary materials**

**Table S1**. Characteristics of patients with respiratory bacteria sequencing (N = 52)

| Characteristics | COPD |
| --- | --- |
| N | 52 |
| Age(years), Mean±SD | 66.73±7.59 |
| Gender (Male), n (%) | 47(90.4%) |
| BMI (kg/m^2^), Mean±SD | 23.02±3.03 |
| Smoking status, n (%) | |
| Current smokers | 24(46.2%) |
| Non-smokers | 28(53.8%) |
| Smoking pack years, n (%) | |
| ≤20 | 12(23.1%) |
| 20<n≤40 | 21(40.4%) |
| >40 | 19(36.5%) |
| ICS treatment, n (%) | |
| Yes | 23(44.2%) |
| No | 29(55.8%) |
| Exacerbation in the past year, n (%) | |
| Yes | 23(44.2%) |
| No | 29(55.8%) |
| mMRC score, Median (IQR) | 2(1) |
| CAT score, Median (IQR) | 11.5(5) |
| Spirometry tests, Mean±SD | |
| FEV1(L) | 1.40±0.48 |
| FEV1%pred | 49.40±17.34 |
| FVC(L) | 2.67±0.61 |
| FVC%pred | 73.09±16.14 |
| FEV1/FVC | 52.01±11.00 |
| DLco SB%pred | 70.80±28.25 |
| DLco/VA%pred | 76.13±26.76 |
| GOLD grade, n (%) | |
| 1 | 2(3.8%) |
| 2 | 20(38.5%) |
| 3 | 24(46.2%) |
| 4 | 6(11.5%) |
| GOLD group, n (%) | |
| A | 14(26.9%) |
| B | 17(32.7%) |
| C | 2(3.8%) |
| D | 19(36.5%) |

ICS: inhaled corticosteroids, mMRC: Modified British Medical Research Council Questionnaires, CAT: COPD Assessment Test Questionnaires, FEV1: forced expiratory volume in one second, FEV1%pred: FEV1 as a percentage of the predicted value, FVC: forced vital capacity, FVC%pred: FVC as a percentage of the predicted value, FEV1/FVC: the ratio of FEV1 to FVC, DLco SB: diffusing capacity of the lungs for carbon monoxide obtained by the single breath holding technique, DLco SB%pred: DLco SB as a percentage of the predicted value, DLco/VA: diffusing capacity for carbon monoxide per unit of alveolar volume, DLco/VA%pred: DLco/VA as a percentage of the predicted value.

**Table S2.** Comparison of clinical characteristics and pulmonary function between *Streptococcus* cluster and *Rothia* cluster.

| Characteristics | *Streptococcus* cluster（n=33） | *Rothia* cluster  （n=19） | Unpaired t-test / Mann-Whitney U test/chi-square test (Fisher's exact test) | |
| --- | --- | --- | --- | --- |
|  |  |  | Test statistics | *P*-value |
| Age, Mean±SD | 66.30±6.054 | 67.47±9.851 | *t* = -0.532 | 0.597 |
| Gender (Male), n (%) | 28（84.8%） | 19（100.0%） | χ² = 3.185 | 0.074 |
| BMI, Mean±SD | 23.264±3.044 | 22.583±3.024 | *t* = 0.778 | 0.440 |
| Smoking pack year, Median (IQR) | 40(38.8) | 40(30) | *z* = -1.162 | 0.245 |
| Currently smoking or not, n (%) | | | | |
| Yes | 12(36.4%) | 12(63.2%) | χ² = 3.483 | 0.062 |
| No | 21(63.6%) | 7(36.8%) |  |  |
| ICS treatment, n (%) | | | | |
| Yes | 15(45.5%) | 8(42.1%) | χ² = 0.055 | 0.815 |
| No | 18(54.5%) | 11(57.9%) |  |  |
| Exacerbation occurred in the past year, n (%) | | | | |
| Yes | 18(54.5%) | 5(26.3%) | χ² = 3.895 | 0.048 |
| No | 15(45.5%) | 14(73.7%) |  |  |
| mMRC, Median (IQR) | 2(1) | 2(1) | *z* = -0.275 | 0.783 |
| CAT, Median (IQR) | 10(5) | 12(4) | *z* = -1.805 | 0.071 |
| GOLD grade, n (%) | | | | |
| 1 | 1(3.0%) | 1(3.0%) | / | 0.510 |
| 2 | 15(45.5%) | 5(26.3%) |  |  |
| 3 | 14(42.4%) | 10(52.6%) |  |  |
| 4 | 3(9.1%) | 3(15.8%) |  |  |
| GOLD group n (%) | | | | |
| A | 10(30.3%) | 4(21.1%) | / | 0.136 |
| B | 7(21.2%) | 10(52.6%) |  |  |
| C | 2(6.1%) | 0(0.0%) |  |  |
| D | 14(42.4%) | 5(26.3%) |  |  |
| Spirometry tests, Mean±SD | | | *t* | *P*-value |
| FEV1(L) | 1.448±0.519 | 1.327±0.416 | 0.863 | 0.392 |
| FEV1%pred | 51.585±17.28 | 45.616±17.237 | 1.200 | 0.236 |
| FVC(L) | 2.663±0.649 | 2.684±0.562 | -0.116 | 0.908 |
| FVC%pred | 73.748±14.341 | 71.958±19.249 | 0.382 | 0.704 |
| FEV1/FVC | 53.586±11.270 | 49.275±10.233 | 1.372 | 0.176 |
| DLco SB%pred | 76.233±30.909 | 61.372±20.370 | *t* = 1.871 | 0.067 |
| DLco/VA%pred | 82.911±25.069 | 64.471±26.144 | *t* = 2.515 | 0.015 |

**Table S3**. Characteristics of patients with respiratory fungi sequencing (N = 30)

| Characteristics | COPD |
| --- | --- |
| N | 30 |
| Age(years), Mean±SD | 69.27±7.04 |
| Gender (Male), n (%) | 29 (96.7%) |
| BMI (kg/m^2^), Mean±SD | 22.65±3.20 |
| Smoking status, n (%) | |
| Current smokers | 14(46.7%) |
| Non-smokers | 16(53.3%) |
| Smoking pack years, n (%) | |
| ≤20 | 6(20.0%) |
| 20<years≤40 | 15(50.0%) |
| >40 | 9(30.0%) |
| ICS treatment, n (%) | |
| Yes | 13(43.3%) |
| No | 17(56.7%) |
| Exacerbation occurred in the past year, n (%) | |
| Yes | 10(33.3%) |
| No | 20(66.7%) |
| mMRC score, Median (IQR) | 2(1) |
| CAT score, Median (IQR) | 12(6) |
| Spirometry tests, Mean±SD | |
| FEV1(L) | 1.34±0.42 |
| FEV1%pred | 48.12±17.12 |
| FVC(L) | 2.64±0.53 |
| FVC%pred | 73.21±16.23 |
| FEV1/FVC | 50.56±11.42 |
| DLco SB%pred | 65.01±23.47 |
| DLco/VA%pred | 66.38±22.45 |
| GOLD grade, n (%) | |
| 1 | 2(6.7%) |
| 2 | 9(30.0%) |
| 3 | 16(53.3%) |
| 4 | 3(10.0%) |
| GOLD group, n (%) | |
| A | 6(20.0%) |
| B | 14(46.7%) |
| C | 1(3.3%) |
| D | 9(30.0%) |

**Table S4.** Comparison of clinical characteristics and pulmonary function between *Aspergillus* cluster and *Candida* cluster.

| Characteristics | *Aspergillus* cluster（n=17） | *Candida* cluster（n=13） | Unpaired t-test / Mann-Whitney U test/ Fisher's exact test | |
| --- | --- | --- | --- | --- |
|  |  |  | Test statistics | *P*-value |
| Age, Mean±SD | 68.94±8.17 | 69.69±5.51 | *t* = -0.285 | 0.778 |
| Gender (Male), n (%) | 17（100%） | 12（92.3%） | / | 0.433 |
| BMI, Mean±SD | 23.002±3.01 | 22.20±3.50 | *t* = 0.675 | 0.505 |
| Smoking pack year, M(IQR) | 40(28.8) | 40(15.0) | *z* = -0.468 | 0.640 |
| Currently smoking or not, n (%) | | | | |
| Yes | 8(47.1%) | 6(46.2%) | / | 1.000 |
| No | 9(52.9%) | 7(53.8%) |  |  |
| ICS treatment, n (%) | | | | |
| Yes | 6(35.3%) | 7(53.8%) | / | 0.460 |
| No | 11(64.7%) | 6(46.2%) |  |  |
| Exacerbation occurred in the past year, n (%) | | | | |
| Yes | 7(41.2%) | 3(23.1%) | / | 0.440 |
| No | 10(58.8%) | 10(76.9%) |  |  |
| mMRC, M(IQR) | 2(1) | 2(2) | *z* = -1.327 | 0.184 |
| CAT, M(IQR) | 13(6) | 12(5) | *z* = -1.746 | 0.081 |
| GOLD grade, n (%) | | | | |
| 1 | 2(11.8%) | 0(0%) | / | 0.027 |
| 2 | 2(11.8%) | 7(53.8%) |  |  |
| 3 | 10(58.8%) | 6(46.2%) |  |  |
| 4 | 3(17.6%) | 0(0%) |  |  |
| GOLD group n (%) | | | | |
| A | 2(11.8%) | 4(30.8%) | / | 0.207 |
| B | 8(47.1%) | 6(46.2%) |  |  |
| C | 0(0%) | 1(7.7%) |  |  |
| D | 7(41.2%) | 2(15.4%) |  |  |
| Spirometry tests, Mean±SD | | | *t* |  |
| FEV1(L) | 1.20±0.41 | 1.52±0.36 | -2.298 | 0.029 |
| FEV1%pred | 42.85±19.52 | 55.02±10.48 | -2.029 | 0.052 |
| FVC(L) | 2.55±0.53 | 2.76±0.53 | -1.054 | 0.301 |
| FVC%pred | 69.61±17.96 | 77.92±12.80 | -1.415 | 0.168 |
| FEV1/FVC | 46.87±12.05 | 55.39±8.77 | -2.149 | 0.040 |
| DLco SB%pred | 59.26±19.48 | 72.53±26.77 | -1.574 | 0.127 |
| DLco/VA%pred | 60.99±22.92 | 73.44±20.55 | -1.541 | 0.135 |

**Table S5.** Comparison of the radiomics features in patients of two bacterial clusters.

| Measured indicators（Mean±SD） | *Streptococcus* cluster（n=17） | *Rothia* cluster  （n=11） | 95% IC of difference | Unpaired t-test | |
| --- | --- | --- | --- | --- | --- |
|  |  |  |  | *t* | *P*-value |
| LAA% (%) | | | | | |
| Whole lung | 29.36±13.54 | 39.49±9.98 | 0.352,19.912 | 2.130 | 0.043 |
| Right upper lobe | 31.15±14.77 | 40.42±13.45 | -2.082,20.625 | 1.678 | 0.105 |
| Right middle lobe | 31.70±15.54 | 41.28±9.59 | -1.206, 20.370 | 1.826 | 0.079 |
| Right lower lobe | 27.77±14.98 | 37.06±10.07 | -1.288,19.874 | 1.805 | 0.083 |
| Left upper lobe | 29.66±15.08 | 42.03±10.96 | 1.525,23.227 | 2.344 | 0.027 |
| Left lower lobe | 27.78±14.35 | 37.79±9.93 | -0.193,20.221 | 2.017 | 0.054 |
| WT/$\sqrt{\mathrm{BSA}}$ | | | | | |
| 3^rd^ generation | 0.93±0.28 | 1.16±0.27 | 0.147,0.451 | 2.194 | 0.037 |
| 4^th^ generation | 0.72±0.19 | 0.89±0.25 | 0.002,0.345 | 2.074 | 0.048 |
| 5^th^ generation | 0.65±0.15 | 0.74±0.17 | -0.042,0.210 | 1.368 | 0.183 |
| 6^th^ generation | 0.59±0.09 | 0.67±0.12 | 0.002,0.162 | 2.105 | 0.045 |
| Ai/BSA | | | | | |
| 3rd generation | 37.35±16.72 | 29.03±9.00 | -1.717, 8.366 | 1.707 | 0.100 |
| 4th generation | 19.17±9.64 | 19.09±8.02 | -7.115, 7.277 | 0.023 | 0.983 |
| 5th generation | 10.49±5.94 | 11.92±765 | -6.716, 3.866 | -0.554 | 0.585 |
| 6th generation | 7.38±4.70 | 7.96±4.98 | -4.400, 3.248 | -0.310 | 0.759 |

**Table S6.** Comparison of the radiomics features in patients of two fungal clusters.

| Measured indicators（Mean±SD） | *Aspergillus* cluster  （n=7） | *Candida* cluster  （n=8） | 95% IC of difference | Unpaired *t*-test | |
| --- | --- | --- | --- | --- | --- |
|  |  |  |  | *t* | *P*-value |
| LAA% (%) | | | | | |
| Whole lung | 39.75±11.26 | 31.69±12.91 | -6.86, 23.00 | 1.168 | 0.264 |
| Right upper lobe | 44.91±10.37 | 30.44±15.52 | -0.50, 29.45 | 2.089 | 0.057 |
| Right middle lobe | 39.94±13.48 | 34.41±13.35 | -9.46, 20.52 | 0.797 | 0.440 |
| Right lower lobe | 33.61±14.07 | 30.03±14.73 | -12.55, 19.73 | 0.481 | 0.639 |
| Left upper lobe | 45.24±9.90 | 33.23±15.19 | -2.55, 26.56 | 1.781 | 0.098 |
| Left lower lobe | 35.37±12.37 | 31.00±15.95 | -11.74, 20.48 | 0.586 | 0.568 |
| WT/$\sqrt{\mathrm{BSA}}$ | | | | | |
| 3rd generation | 1.04±0.31 | 1.11±0.24 | -0.37, 0.24 | -0.460 | 0.653 |
| 4th generation | 0.90±0.31 | 0.75±0.16 | -0.11, 0.43 | 1.253 | 0.232 |
| 5th generation | 0.74±0.21 | 0.65±0.13 | -0.11, 0.29 | 0.988 | 0.341 |
| 6th generation | 0.61±0.12 | 0.64±0.15 | -0.19, 0.12 | -0.459 | 0.654 |
| Ai/BSA | | | | | |
| 3rd generation | 25.52±9.53 | 38.26±11.25 | -24.47, -1.00 | -2.345 | 0.036 |
| 4th generation | 16.76±11.23 | 21.94±6.75 | -15.35, 4.99 | -1.101 | 0.291 |
| 5th generation | 10.32±9.17 | 12.00±4.94 | -9.74,6.37 | -0.451 | 0.659 |
| 6th generation | 7.65±6.29 | 8.29±4.83 | -6.85, 5.57 | -0.222 | 0.828 |


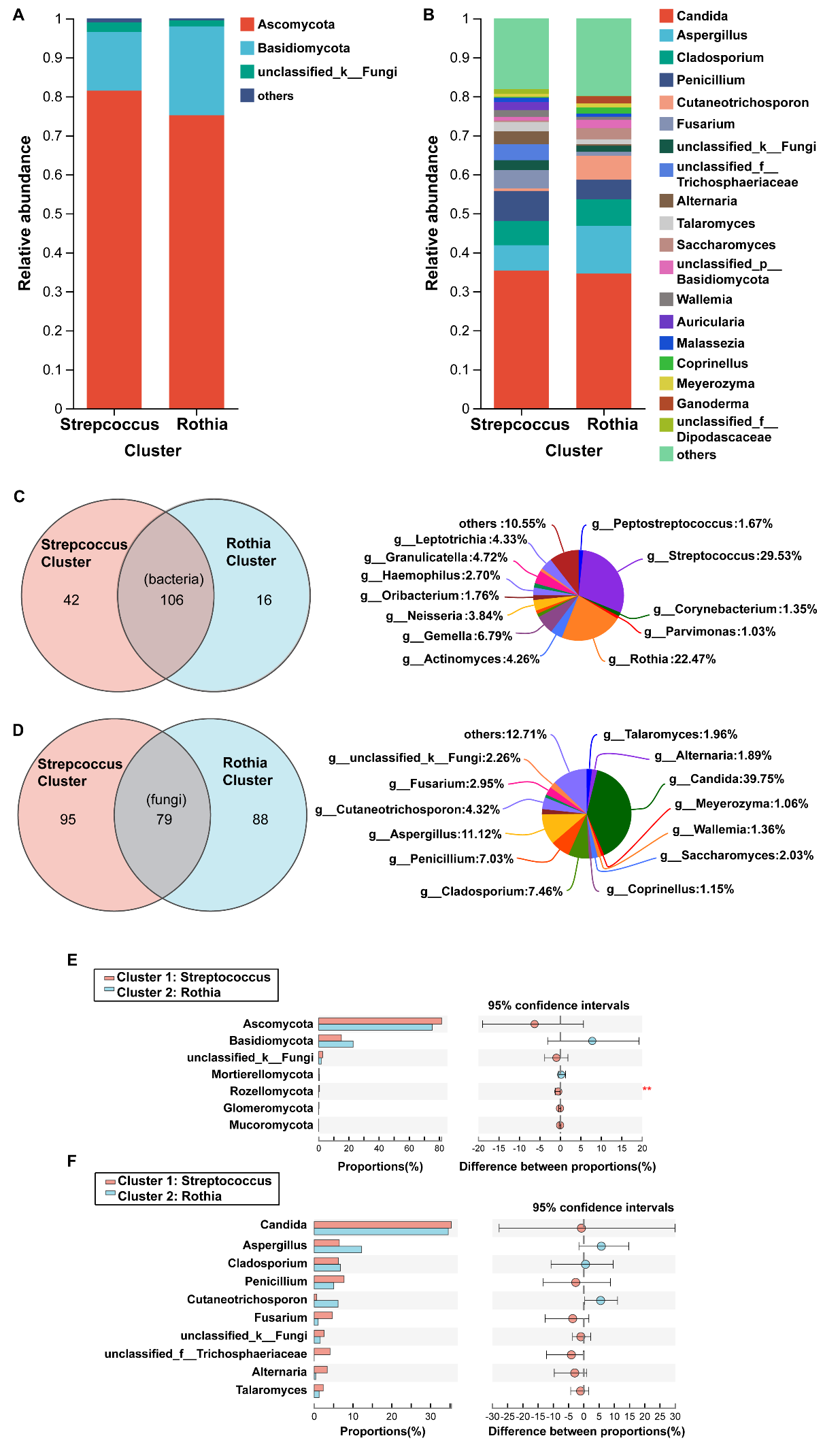


**Fig S1**. **Fungi in the *Streptococcus* and *Rothia* clusters**. A. Fungi of the *Streptococcus* and *Rothia* cluster at the phylum level. B. Fungi of the *Streptococcus* and *Rothia* cluster at the genera level. C. Venn diagram showed shared and specific bacteria at the genera level between *Streptococcus* and *Rothia* cluster and percentage of major shared bacteria. D.Venn diagram showed shared and specific fungi at the genera level between *Streptococcus* and *Rothia* cluster and percentage of major shared fungi. E. Significantly differing fungi between *Streptococcus* and *Rothia* cluster at the phylum level. F. No significant differences in fungi at the genera level between *Streptococcus* and *Rothia* cluster. (* 0.01≤ FDR *P* <0.05, ** 0.001≤ FDR *P* < 0.01, *** FDR *P* < 0.001).


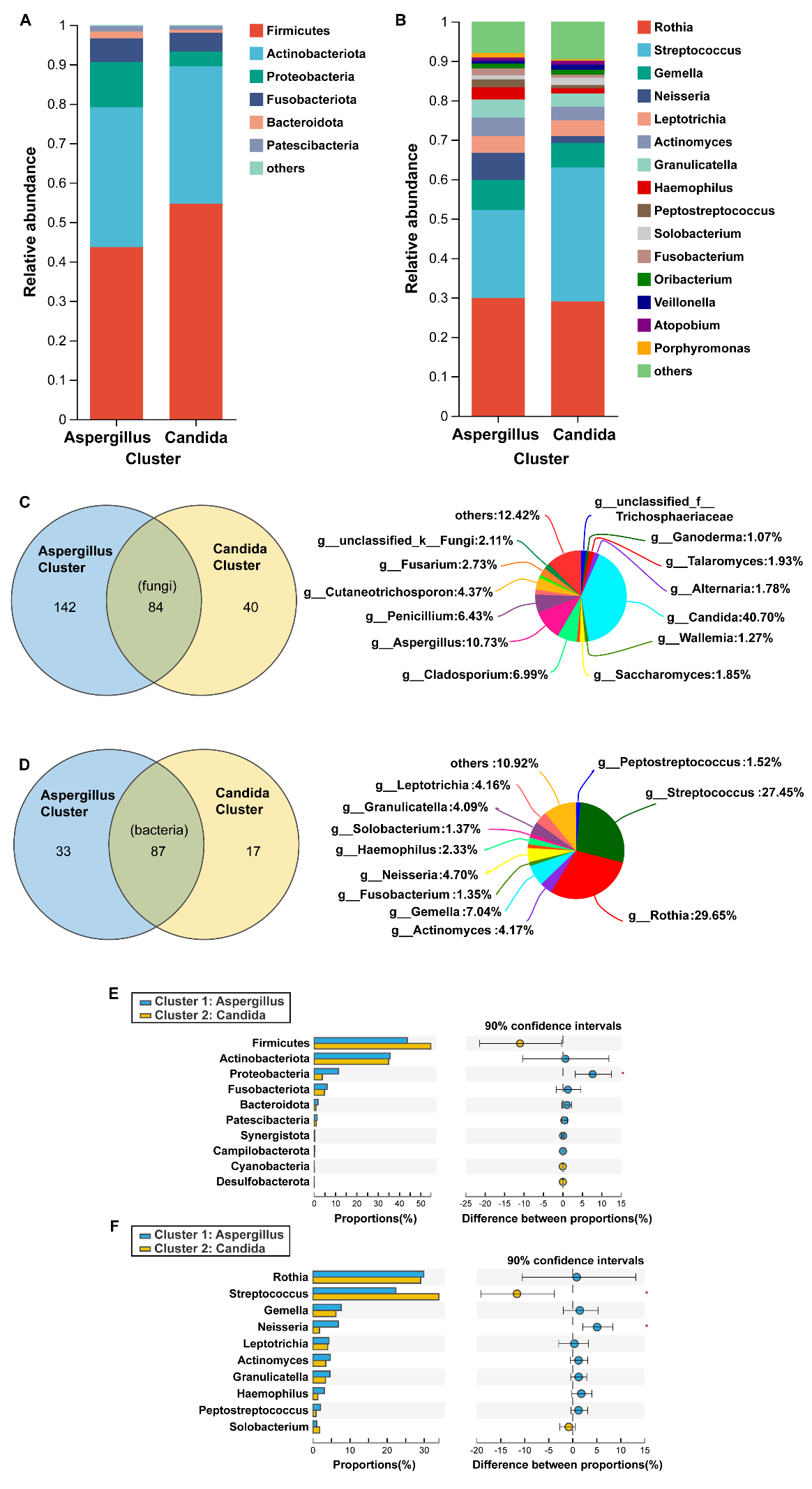


**Fig S2. Bacteria in the *Aspergillus* and *Candida* clusters.** A. Bacteria of the *Aspergillus* and *Candida* cluster at the phylum level. B. Bacteria of the *Aspergillus* and *Candida* cluster at the genera level. C. Venn diagram showed shared and specific fungi at the genera level between *Aspergillus* and *Candida* cluster and percentage of major shared fungi. D. Venn diagram showed shared and specific bacteria at the genera level between *Aspergillus* and *Candida* cluster and percentage of major shared bacteria. E. Significantly differing bacteria between *Aspergillus* and *Candida* cluster at the phylum level. F. Significantly differing bacteria between *Aspergillus* and *Candida* cluster at the genera level. (* 0.01≤ FDR *P* <0.05, ** 0.001≤ FDR *P* < 0.01, *** FDR *P* < 0.001)


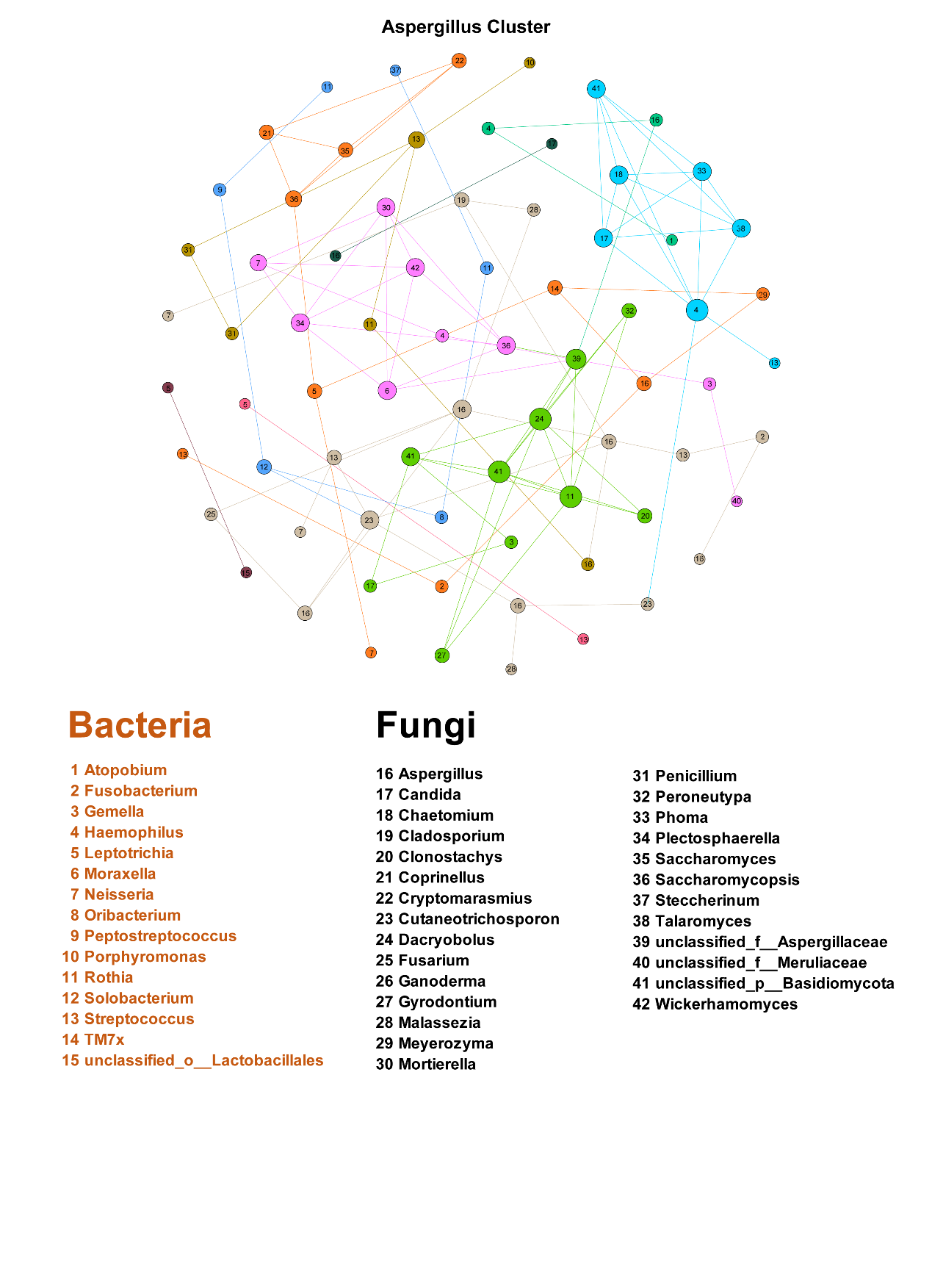


**Fig S3. Bacteria– fungi, bacteria– bacteria, and fungi– fungi connections in *Aspergillus* cluster.** Fungal nodes and bacterial nodes are labeled in different numbers according to the modules. Nodes are colored by modules or microbial communities at a resolution of 0.8 using the modularity function in Gephi software. Networks represent statistically significant correlations (FDR *P* < 0.05).


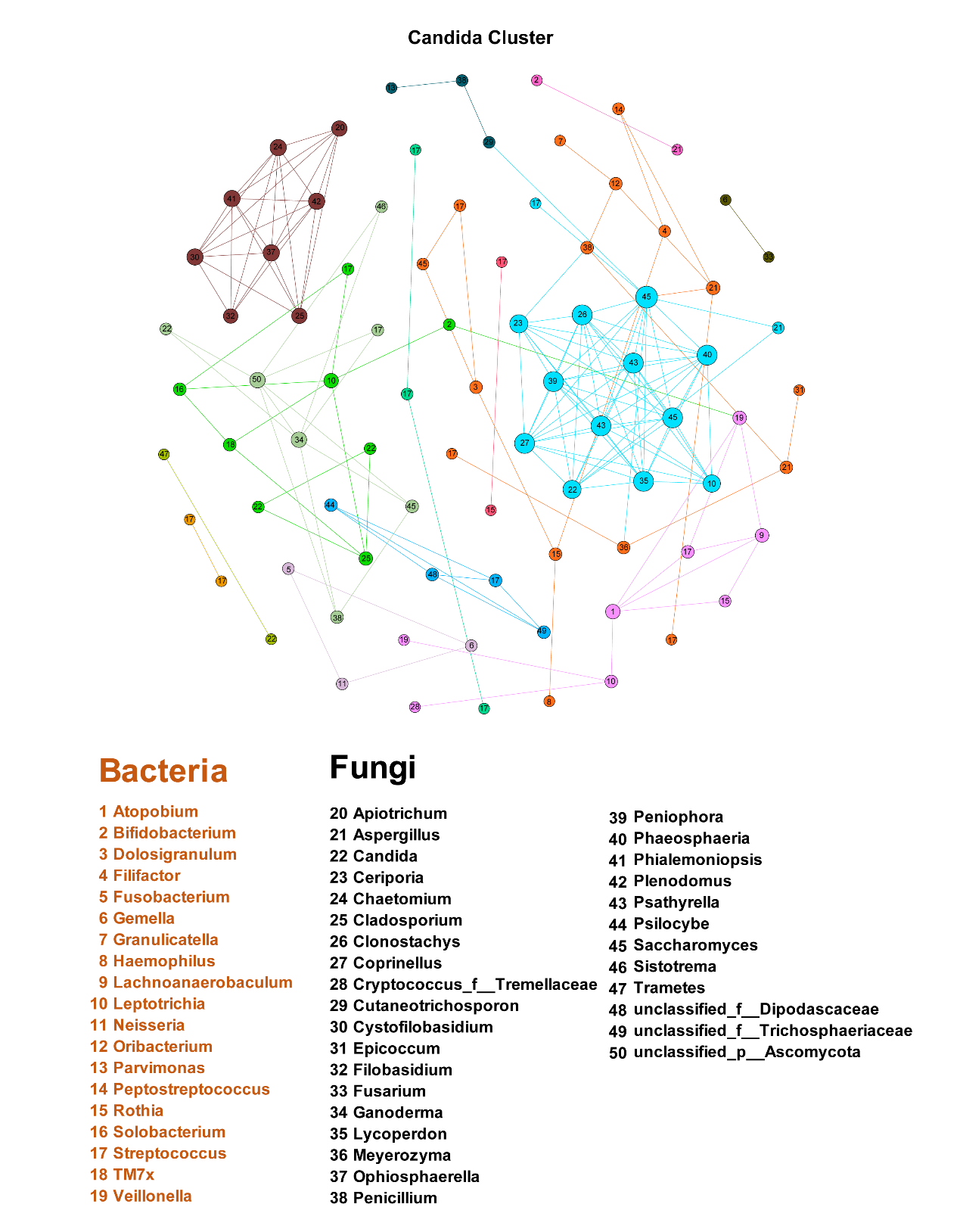


**Fig S4. bacteria– fungi, bacteria– bacteria, and fungi– fungi connections in Candida cluster.** Fungal nodes and bacterial nodes are labeled in different numbers according to the modules. Nodes are colored by modules or microbial communities at a resolution of 0.8 using the modularity function in Gephi software. Networks represent statistically significant correlations (FDR *P* < 0.05).
